# Isolation and characterization of a novel hydrolase-producing probiotic *Bacillus licheniformis* and its application in the fermentation of soybean meal

**DOI:** 10.3389/fnut.2023.1123422

**Published:** 2023-03-08

**Authors:** Nanshan Qi, Xiaoshu Zhan, Joshua Milmine, Maureen Sahar, Kai-Hsiang Chang, Julang Li

**Affiliations:** ^1^Institute of Animal Health, Guangdong Academy of Agricultural Sciences, Guangzhou, Guangdong, China; ^2^Department of Animal Biosciences, University of Guelph, Guelph, ON, Canada; ^3^Department of Life Science and Engineering, Foshan University, Foshan, Guangdong, China

**Keywords:** soybean meal, *Bacillus licheniformis*, phytase, solid-fermentation, probiotic

## Abstract

Soybean meal (SBM) is one of the most important sources of plant-based protein in the livestock and poultry industry. However, SBM contains anti-nutritional factors (ANFs) such as glycinin, β-conglycinin, trypsin inhibitor and phytic acid that can damage the intestinal health of animals, inevitably reducing growth performance. Fermentation using microorganisms with probiotic potential is a viable strategy to reduce ANFs and enhance the nutritional value of SBM. In this study, a novel potential probiotic *Bacillus licheniformis* (B4) with phytase, protease, cellulase and xylanase activity was isolated from camel feces. The ability of B4 to tolerate different pH, bile salts concentrations and temperatures were tested using metabolic activity assay. It was found that B4 can survive at pH 3.0, or 1.0% bile salts for 5 h, and displayed high proliferative activity when cultured at 50°C. Furthermore, B4 was capable of degrading glycinin, β-conglycinin and trypsin inhibitor which in turn resulted in significant increases of the degree of protein hydrolysis from 15.9% to 25.5% (*p* < 0.01) and crude protein from 44.8% to 54.3% (*p* < 0.001). After fermentation with B4 for 24 h, phytic acid in SBM was reduced by 73.3% (*p* < 0.001), the neutral detergent fiber (NDF) and the acid detergent fiber of the fermented SBM were significantly decreased by 38.40% (*p* < 0.001) and 30.20% (*p* < 0.05), compared to the unfermented SBM sample. Our results suggested that the effect of solid-state fermented SBM using this novel *B. licheniformis* (B4) strain, could significantly reduce phytic acid concentrations whilst improving the nutritional value of SBM, presenting itself as a promising alternative to phytase additives.

## Introduction

Soybean meal (SBM), contains 40%–50% crude protein and is one of the most important sources of supplemental plant-based protein in the livestock and poultry industry (1, 2). However, anti-nutritional factors (ANFs) such as allergenic proteins, glycinin (22–40 kDa) and β-conglycinin (52–76 kDa) found within SBM can damage the intestinal structure of young pigs and chickens, leading to poor nutrient absorption and increased incidence of diarrhea, which in turn results in poor growth performance (3–7). Phytic acid is a feed nutrition inhibitor that can chelate micronutrients resulting in the formation of insoluble cation-phytic acid complexes which in turn leads to decreased absorption of various cations (calcium, iron and zinc) in the intestine of animals. Trypsin inhibitor (TI, 20 kDa) can reduce the protein digestion and restrict the absorption and utilization of nutrients (8). In addition to this, undigested proteins can be consumed by potential pathogen bacteria that work to impair the integrity of the intestinal membrane (9).

Enzymatic hydrolysis technology has been used to reduce allergenicity and improve the nutritional value of SBM. Supplementation of protease derived from *Aspergillus* or *Bacillus* strains can reduce the allergenic proteins in SBM significantly (10, 11). Phytase, xylanase or cellulase derived from fungi and bacteria can improve the digestibility of plant-based feed ingredients, decrease intestinal content viscosity and enhance nutrient absorption which in turn modulates the intestinal health of animals (12). However, the stability of enzymatic activity and the high cost of enzyme preparations are the primary factors limiting their application in livestock feeds.

Fermentation by probiotics is a new strategy that has been deployed to reduce immunoreactivity and enhance the nutritional value of SBM. Previous research has performed SBM fermentation using *Lactobacillus* spp., *Bacillus* spp., *Lactiplantibacillus* spp., *Enterococcus* spp., *Pediococcus* spp. to decrease the ANFs and increase the protein contents (13, 14). Some strains of probiotics are known to produce enzymes such as phytase, xylanase or cellulase, which help to reduce the allergenicity and improve the nutrient digestibility of SBM. These strains can be seen as an alternative to enzyme additives for supplementation (3). The optimal temperature and reduction of undesired microorganisms’ contamination (such as *Escherichia coli*) are the key indicators of a high quality and stable fermentation. For example, the high temperatures (up to 50°C–60°C) produced by fermentation can decrease the growth of undesired contaminated bacterial, and also inhibit the growth of most probiotic strains; therefore resulting in an inefficient fermentation (15). However, some studies showed that the use of sterilized SBM, as substrate for fermentation, could reduce undesired bacterial contamination and result in a better outcome when fermented (16, 17). Although this strategy provides an improvement in the high-quality fermentation of SBM, complicated processes and high energy consumption limits its viability in industrial SBM fermentation. Therefore, a strain of bacteria that is capable of enzyme secretion, has a high tolerance to a broad spectrum of varying temperatures, and is able to inhibit the growth of contaminated bacteria would be desirable to use for fermentation to improve the nutritional value of SBM.

Previous studies have shown that *Bacillus licheniformis* (*B. licheniformis*) improve growth performance in both swine and broilers when used as probiotic (5, 18). However, little is known about the efficiency of enzyme production and the function of SBM fermentation by *B. licheniformis*. In this study, we isolated a thermophilic enzyme-producing (phytase, protease, cellulase and xylanase) strain of *B. licheniformis* from camel feces and demonstrate its potential for the solid-state fermentation of SBM.

## Materials and methods

### Chemicals and media

Unless specified otherwise, all chemicals and media were purchased from Fisher Scientific, Mississauga, Canada.

### Isolation and identification of *Bacillus licheniformis* from camel feces

*Bacillus licheniformis* was isolated from camel feces using a protocol described by Akhtar et al. (3) with minor modifications. The camel fecal samples were resuspended in sterile PBS (10 mL), vortexed for 10 s in order to suspend and collect the fecal supernatant. The collected supernatant was then diluted 10 times with PBS. Samples were centrifuged for 2 min at 400 × g and 100 μL of the original sample and 10-times diluted fecal suspension were plated on Luria-Bertani (19) agar plates and incubated at 37°C for 24 h. Thirty-seven bacterial colonies with different morphologies were purified using a streak plate method, and the purified bacteria were submitted to the Animal Health Laboratory at the University of Guelph for species verification using a matrix-assisted laser desorption ionization-time of flight mass spectrometry (MALDI-TOF MS, Bruker, Canada). Five isolates of *B. licheniformis* (tentatively named A4, A11, A16, B2, and B4) were selected for phytase analysis.

### Screening for phytase-producing *Bacillus licheniformis*

Western blot (WB) was performed to screen the phytase-producing *B. licheniformis*. The five isolates of *B. licheniformis* (tentatively named A4, A11, A16, B2, and B4) were cultured in LB broth under 37°C at 200 revolutions per minute (RPM) for 24 h. Cell-free culture supernatant (CFCS) was obtained after centrifugation (5,000 × g, 15 min) for further western blot analysis. Twenty micrograms (total protein concentration) of CFCS were loaded into an 11% SDS PAGE gel. After gel electrophoresis, the proteins were transferred to a polyvinyl difluoride (PVDF) membrane (Millipore, Billerica, United States) using the Trans-BlotR Turbo™ transfer system (Biorad, United States). Membranes were blocked in TBS-T with 5% skim milk powder at room temperature for 1 h and were incubated with the mouse anti-phytase primary antibody (1:1,000 dilution, Artron, Canada) at 4°C, overnight. The anti-mouse IgG (1:5,000 dilution, HRP-linked; Cell Signaling Technology; United States) was used as the secondary antibody. The protein density was detected by the Clarity Western ECL substrate and imaged with the ChemiDoc XRS+ System (Bio-Rad, United States).

### Detection of protease, cellulase, and xylanase activities of *Bacillus licheniformis* (B4)

The agar plates containing allergenic proteins (Glycinin/β-conglycinin), carboxymethyl cellulose (CMC) (Acros Organics, United States) and Xylan (Sigma Aldrich, United States) were used to detect the protease, cellulase and xylanase activities of B4 according to the previous study by Li et al. (20),with minor modifications. SBM (Floradale Feed Mill Ltd., Floradale, Ontario, Canada) was grinded and filtered through a 60-mesh sieve. Five grams of ground SBM was suspended with 75 ml 0.03 M Tris–HCl (pH 8.5) and stirred at 200 RPM for 1 h at 45°C followed by centrifuging at 9,000 × g for 40 min at 4°C. After centrifugation, the supernatant was transferred to a new container for analysis and an additional precipitate pellet was performed with the aforementioned procedure described above for extraction To obtain the glycinin, NaHSO_3_ (final concentration 10 mM) was added to the supernatant and adjusted to pH of6.4 with 2 M hydrochloric acid (HCl). After storing the mixture at 4°C overnight, the glycinin-rich fraction of precipitate was obtained after centrifugation at 6,500 × g for 30 min at 4°C. To further extract β-conglycinin, NaCl (final concentration 0.25 M) was added to the supernatant and adjusted to pH of 5.5 with 2 M HCl, then stirred at 200 RPM for 30 min at 45°C followed by centrifuging at 9,000 × g for 40 min at 4°C. After centrifugation, the supernatant was transferred to a new collection tube and diluted by two-fold with Milli-Q water (MQ water) and adjusted to a pH of 4.8 with 2 M HCl. The β-conglycinin-rich fraction of precipitate was then obtained after centrifugation at 6,500 × g for 20 min at 4°C. The glycinin or β-conglycinin precipitate was dissolved in 100 ml of MQ water, and was supplemented with an equal volume of LB broth and 1.5 g agar to prepare glycinin or β-conglycinin protein plates. The plates were then prepared by adding 15 mL of glycinin or β-conglycinin protein broth and 15 mL of LB broth. The CMC agar plates were prepared by dissolving 1 g of CMC and 1.5 g of agar in 100 mL of LB. The Xylan agar plates were prepared by dissolving 0.3 g of Xylan and 1.5 g of agar to 100 ml of LB. The broth used was autoclaved for 25 min at 121°C before pouring into the petri dishes.

A single colony of *B. licheniformis* (B4) or *Bacillus cereus* (negative control) was cultured in LB broth overnight at 37°C, and 1 μL of B4 culture broth was inoculated on the agar plates containing allergenic proteins (Glycinin/β-conglycinin), CMC or Xylan, respectively. The clear zone diameters around the colonies were recorded after 12 h of culturing at 37°C and the hydrolysis capacity (HC) was calculated as the ratio of the diameter of the clear zone (HD) and colony (CD). Each experiment was performed in triplicate.

### Growth characteristics of bacteria at different pH, bile salts, and temperature

The tolerance of *B. licheniformis* (B4) in low acidic, high bile salts, and high temperature environments was assessed according to the method reported in a previous study by Sudan et al. (21) with minor modifications. Briefly, for the acid tolerance and bile salts assay, 30 μL of the B4 overnight cultures was mixed with 70 μL of LB broth which has been adjusted to a pH of 2, 3, and 6.8 (control) using 2 M HCl, or 70 μL of LB broth which contained 0% (control), 0.3, 0.5 and 1% of bile salts (Sigma, United States) in a 96-well microplate for 1, 3, and 5 h at 37°C. At the end of each incubation period, the bacterial viability was measured spectrophotometrically using the Bacterial Counting Colorimetric Assay Kit (Bio Vision Technologies, United States) following manufacturer protocol. The optical density (OD)at 460 nm was then read via a Cytation 5 multimode plate reader (Biotek, Winooski, VT, United States). For temperature tolerance of the B4 in LB broth (pH 6.8), 60 μL of the B4 overnight cultures were incubated with 6 mL of LB broth at 25°C, 37°C, 50°C, or 60°C for 12 h and an OD at 600 nm was read every 2 h over a period of 2–12 h at 37°C using a Cytation 5 multimode plate reader (Biotek, Winooski, VT, United States). Each experiment was performed in triplicate.

### Solid-state fermentation of SBM

*Bacillus licheniformis* (B4) was prepared by incubating a single colony in LB broth at 37°C (200 RPM) for 16 h and diluted to a cell number of 2.0 × 10^7^ CFU/mL to use as inoculum. Thirty grams of SBM were mixed with 30 ml of inoculum (2.0 × 10^7^ CFU/mL) in an aluminum box. The mixture was then incubated at 37°C for 24 or 48 h. To determine if B4 has the antimicrobial activity to eliminate gram-negative bacteria during fermentation, 1 gram of the 24-h fermentation sample was collected and mixed with 10 ml of sterilized MQ water. The samples were then vortexed for 30 s, and 100 μL of re-suspended solution were spread on the MacConkey agar (Fisher Scientific, Mississauga, Canada) plates and cultured at 37°C for 16 h. SBM fermented with MQ water for 24 h was performed the sampling procedure as control. The fermented SBM (F-SBM) samples or unfermented SBM (UF-SBM, control) were dried at 60°C for 24 h after fermentation and grinded to pass through a 60-mesh screen for further ANFs or nutritional value analysis. Each experiment was performed in triplicate.

### Anti-nutritional factors analysis of unfermented and fermented SBM

Allergenic proteins (glycinin and β-conglycinin) and trypsin inhibitors were analyzed according to the method reported in a previous study by Medeiros et al. (22) with minor modifications. Briefly, 0.5 g of dried F-SBM or UF-SBM was suspended in 5 ml of pre-cooled PBS and each sample was ultra-sonicated for 30 s. Samples were then centrifuged at 7,000 × g for 10 min at 4°C and 20 μg of the supernatant from each sample was loaded to 14% sodium-dodecyl sulfate-polyacrylamide gel electrophoresis (SDS-PAGE) in order to analyze the fractionation of soluble proteins. The images were taken using ChemiDoc XRS+ (Bio-Rad, United States) and analyzed with the Image Lab software. Furthermore, WB was performed to determine the pig plasma’s immunoreactivity against the UF-SBM or F-SBM and the digestion of the anti-nutrition factor TI. A WB was accomplished by using pig serum (1: 500 dilution) or Rabbit anti-Trypsin inhibitor antibody (1: 2,000 dilution, ab34549, Abcam, Cambridge, United Kingdom) as the primary antibody at 4°C overnight, respectively. Pig serum was collected from 8-week-old piglets that were previously exposed to feed containing SBM for 28 days and developed an immune response to SBM allergens. The serum was prepared in the same manner described by Medeiros et al. (22). The Rabbit Anti-pig IgG (1: 5,000, ab6777, Abcam, Cambridge, United Kingdom) or Anti-Rabbit IgG (1:5,000 dilution, HRP-linked; Cell Signaling Technology; United States) was used as secondary antibodies. The protein density was detected by the Clarity Western ECL substrate and imaged with the ChemiDoc XRS+ System (Bio-Rad, United States).

PAC of UF-SBM and F-SBM were determined by the colorimetric method as previously reported by Olukomaiya et al. (23) with minor modifications. One gram of the sample was added to 20 mL of HCl 2.4% (m/V) and mixed at 220 RPM for 16 h at room temperature, followed by centrifuging at 4,000 × g for 10 min at 10°C. The supernatant was then supplemented with 2 g of NaCl and mixed at 350 RPM for 20 min at room temperature. Samples were then settled at 4°C for 60 min, followed by centrifuging at 4,000 × g for 20 min at 10°C. Exactly 10 μL of supernatants from each sample was added into a well in a 96-well plate containing 190 μL MQ water. An equal volume of MQ water was tested as a control, while phytic acid (TCI America™) was used as a standard. Thirty microliters of Wades reagent (0.03%, m/V of Iron (III) chloride hexahydrate with 0.3%, m/V of Sulfosalicylic acid, Thermo Scientific™, United States) were mixed and immediately measured at 500 nm using the Cytation 5 multimode plate reader (Biotek, Winooski, VT, United States).

### Protein hydrolysis analysis assay

The degree of protein hydrolysis (DH) was determined using the o-phthaldialdehyde (OPA) assay method, as described by Nielsen et al. (24) with minor modifications. 200 ml of the OPA working solution was prepared by dissolving 7.62 g of sodium tetraborate decahydrate, 0.2 g of sodium dodecyl sulfate (SDS), 0.176 g of DTT (Dithiothreitol, 99%) and 0.16 g of OPA (97%) in MQ water. 0.16 g of OPA was then dissolved in 4 ml of ethanol in advance. To obtain the free amino acid, 0.5 g of UF-SBM or SBM samples were mixed into 10 ml of MQ water, stirred at 200 RPM for 1 h and then centrifuged at 14,000 × g for 20 min at room temperature. Additionally, 0.5 g of UF-SBM or SBM samples were mixed with 6 M of HCl at 110°C for 24 h to completely hydrolyze the total amount of amino acid as a control. Both free amino acid and total amino acid in the supernatant was collected after centrifugation at 1,4000 × g for 20 min at room temperature. A total of 20 μl supernatant of each sample was mixed with 300 μl of the OPA reagent for further absorbance measurements after 2 min of incubation. The absorbance was measured at 340 nm using the Cytation 5 multimode plate reader (Biotek, Winooski, VT, United States) and using MQ water as the control. The DH was then calculated using the formula below.

DH (%) =$\frac{{\mathrm{NH}}_{2}\;\left(\mathrm{free}\right)}{{\mathrm{NH}}_{2}\;\left(\mathrm{total}\right)}$ × 100%, where NH_2_ (free) is the concentration of free amino acid of the samples, and NH_2_ (total) is the total amount of amino acid in the samples after being hydrolyzed in 6 M HCl.

### Chemical analysis of UF-SBM and F-SBM

The dry matter (DM), crude protein (CP), neutral detergent fiber (NDF) and acid detergent fiber (ADF) of the F-SBM were analyzed after 24 or 48 h of solid-fermentation. UF-SBM was used as a control. DM was determined according to the standard operating procedures method 930.15 (Association of Official Analytical Chemists, AOAC, 2005). CP was determined using the DUMAS method (AOAC, 1997) following the manufacturer’s protocol of the LECO Protein/Nitrogen Analyzer (LECO Corp., United States). The NDF and ADF were analyzed with an Ankom2000 Fiber Analyzer (Ankom Technology) following the ANKOM technology procedure 13.

### Statistical analysis

All experiments were conducted in triplicate and the data was analyzed with a one-way analysis of variance (ANOVA) with treatment using a GraphPad Prism software version 9.0. Data was considered significant if *p* < 0.05.

## Results

### Isolation of a novel phytase-producing *Bacillus licheniformis* B4 strain

**Figure 1:**
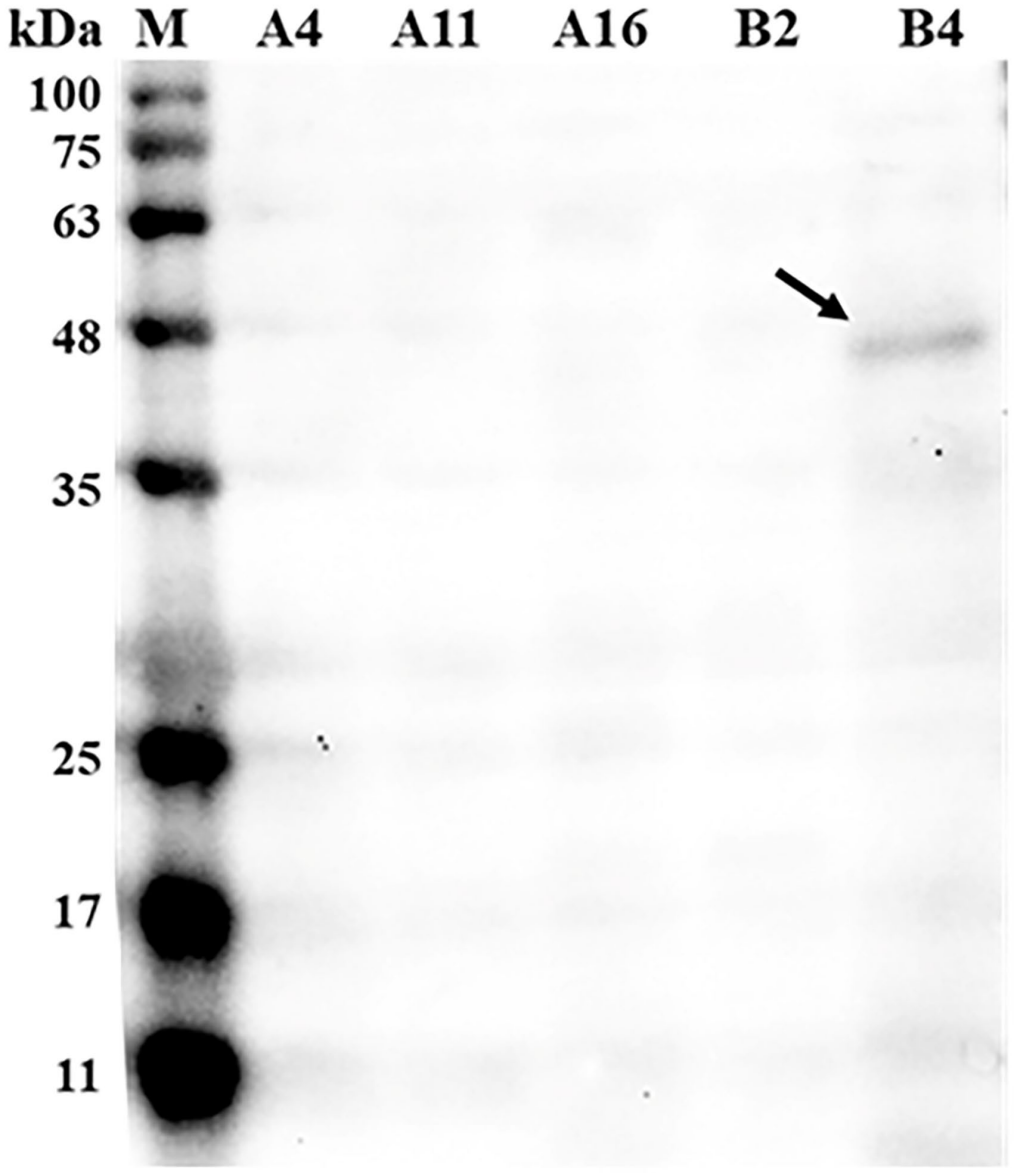
Western blot detecting phytase in the supernatant of bacteria culture broth. Bacterial culture supernatant was loaded into each well at a concentration of 20 μg total protein and incubated with the mouse anti-phytase antibody (Primary antibodies, 1:1,000 dilution) followed by rabbit anti-mouse IgG (secondary antibody, 1:5,000 dilution). M, standard protein markers; A4, *Bacillus licheniformis* A4; A11, *B. licheniformis* A11; A16, *B. licheniformis* A16; B2, *B. licheniformis* B2; B4, *B. licheniformis* B4.

Out of 37 bacterial isolates, five isolates (A4, A11, A16, B2, and B4) were identified as *Bacillus licheniformis* species by the MALDI-TOF MS system. To screen for strains that are capable of phytase secretion, western blot analysis was performed using an antibody against phytase. As shown in Figure 1, anti-phytase antibodies were able to bind to protein (43 kDa) found in the B4 supernatant culture, while no phytase was detected in the other four strains.

**Figure 2:**
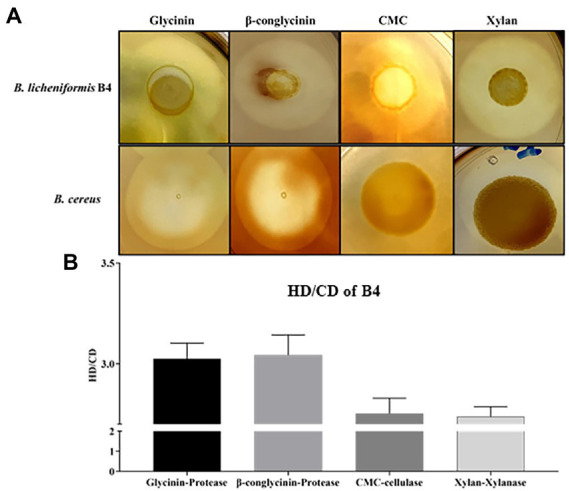
Detection of protease, cellulase, and xylanase activity for *B. licheniformis* B4 using Gtycinin/β-conglycinin, Carboxymethyl cellulose (CMC) and Xylan supplemented agar plates. One microliter of *B. licheniformis* B4 or *B. cereus* (control) overnight culture broth was added on the agar plates containing different allergenic proteins, which was then incubated at 37°C for 12 h. **(A)** Clear zones of *B. licheniformis* B4 or *B. cereus* (negative control) on Glycinin, β-conglycinin, CMC or Xylan supplemented plate. **(B)** Measurement of clear zones of B4. HD. Diameter of clear zone; CD, diameter of colony. Error bars represent the SEM of the mean of three independent observations.

### *Bacillus licheniformis* (B4) secretes protease, cellulase, and xylanase

B4’s protease, cellulase and xylanase activity are shown in Figure 2. Large clear zones occurred on the glycinin, β-conglycinin, CMC or Xylan supplemented agar plates (Figure 2A), in which their HC (HD/CD) values were 3.03 ± 0.082, 3.04 ± 0.10, 2.75 ± 0.08 and 2.74 ± 0.05, respectively, (Figure 2B), indicating that the strong protease, cellulase and xylanase activity of B4. However, there was no clear zone observed on the glycinin, β-conglycinin and CMC supplemented agar plates but only a relatively small clear zone on the xylan supplemented agar plate that contained the negative control, *B. cereus* (Figure 2A).

### B4 Survives in low pH, high bile salts, and high-temperature environments

**Figure 3:**
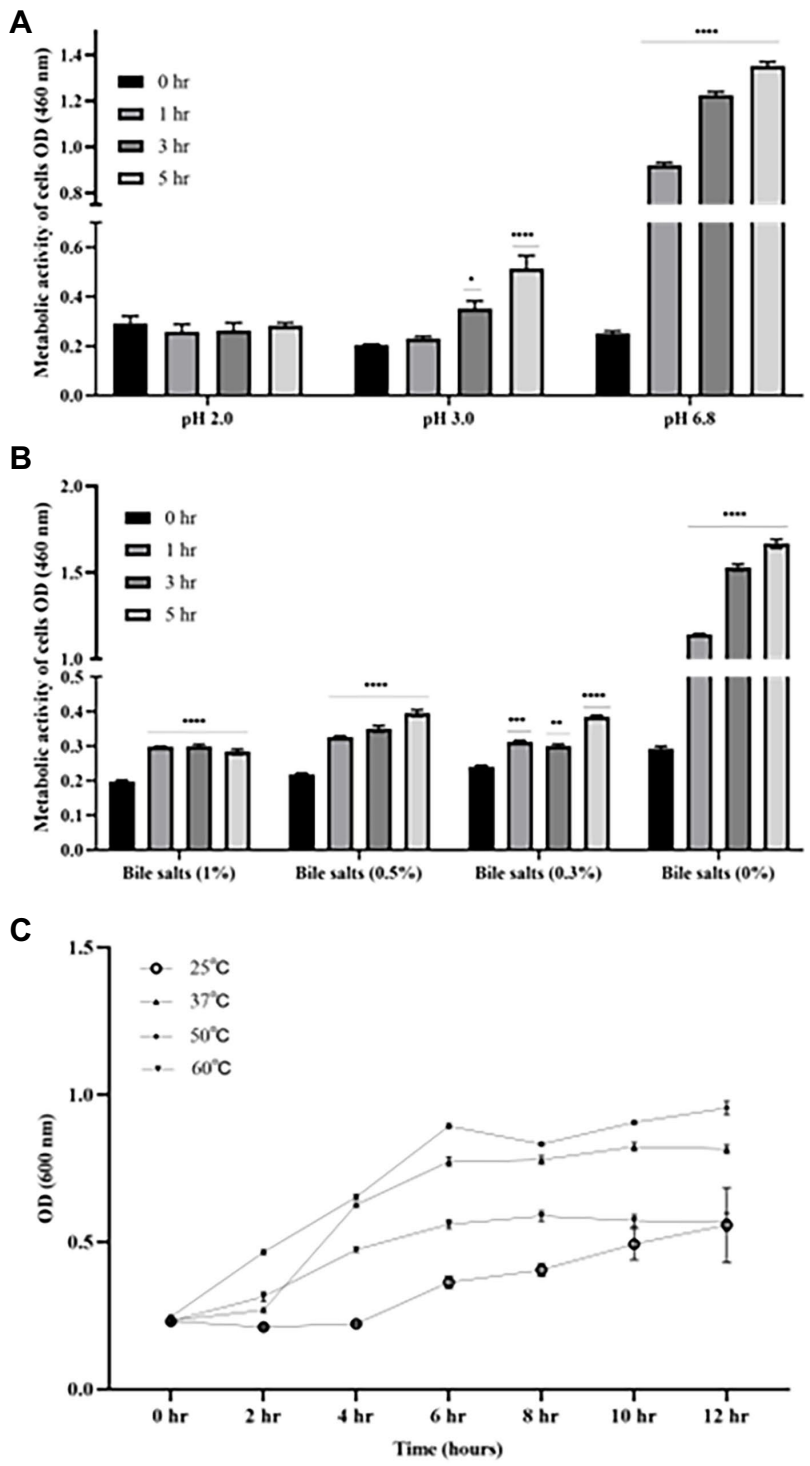
Growth characteristics of B. licheniformis B4 at different pH, Bile salts concentrations, and temperatures. **(A)** Metabolic activity and viability of B4 cells in low pH environment. **(B)** Metabolic activity and viability of B4 cells in high bile salt environment. **(C)** The growth curve of B4 incubated at 25°C, 37°C, 50°C, and 60°C for 12 h. Data are presented as mean ±standard error of the mean (SEM). Bars with statistical significance are denoted as *p < 0.05, **p < 0.01, ***p < 0.005, ****p < 0.0001 using ordinary one-way in analysis of varianve (ANOVA). Significance in all tests was compared with the initial metabolic activity at time zero within each treatment group. All experiments were conducted in triplicate.

As shown in Figure 3, there was no significant difference in the cell activity when incubating B4 in pH of 2.0 for up to 5 h compared to time zero. However, the cell activity of B4 was significantly increased after being after incubated at pH of 3.0 for 3 h (*p* < 0.05), and further increased after 5 h of incubation compared to that of time zero (*p* < 0.001), which suggests that B4 survives in low pH environments. Similarly, the data from the bile salts tolerance assay shows that the growing activity of B4 increased significantly after 1 hour of incubation in the presence of 1% (*p* < 0.001), 0.5% (*p* < 0.001) and 0.3% (*p* < 0.05) of bile salts, when compared to that of time zero (Figure 3B). These results suggested that B4 can easily survive these bile salt concentrations. When examining the growth performance of B4 at various temperatures, cell activities were monitored. It was found that B4 grew well at 37°C and 50°C. The most suitable growth temperature for B4 appears to be 50°C, in which B4 reaches log phase within 2 h and plateaus for 10 h of culturing. Lastly, B4 grows slower at 25°C and 60°C, as compared to 37°C, in which it reaches log phase after 6 h of culturing (Figure 3C).

### Anti-nutritional factors of SBM degradation by solid-state fermentation

**Figure 4:**
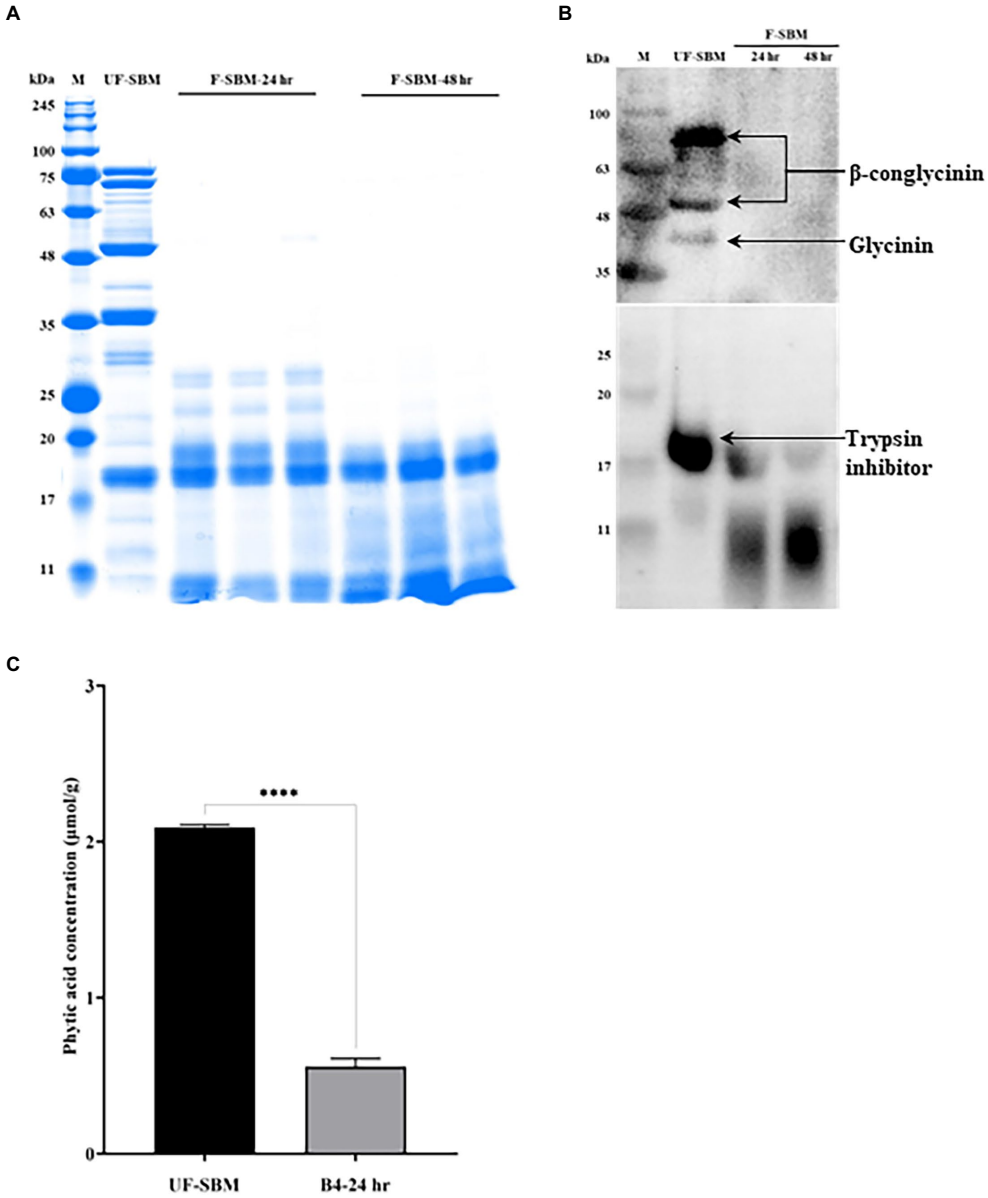
Anti-nutritional factors analysis of unfermented (UF-SBM) and fermented SBM (F-SBM). **(A)** Protein profiling of F-SBM using SDS-PAGE, 20 μg of protein was loaded for each well. Lane 1, Unfermented soybean meal (UF-SBM) was used as control; Lanes 2–4, F-SBM for 24 h; Lanes 5–7, F-SBM for 48 h. **(B)** Western blot detecting soy allergens in the UF-SBM and F-SBM products. 20 μg of protein was loaded for each well and incubated with pig serum (1:500) or anti-trypsin inhibitor antibody (primary antibody, 1:2,000) followed by rabbit anti-piglet IgG (1:5,000) or Anti-rabbit IgG (secondary antibody, 1:5,000). Lane 1, UF-SBM; Lane 2, F-SBM for 24 h; Lane 3, F-SBM for 48 h. **(C)** The Phytic acid (PAC) concentration from 24 h fermentation (B4-24 hr) is relative to the UF-SBM. PAC was determined using the FeCl3 assay method. The absorbance was then measured at 500 nm. Data is presented as mean ±standard error of the mean (SEM). Bars with statistical significance is denoted as ****p < 0.0001 using ordinary one-way in ANOVA. All experiments were conducted in triplicate.

Glycinin (22–40 kDa), β-conglycinin (52–76 kDa) and trypsin inhibitor (20 kDa) are three of the most important allergenic proteins in the SBM. The results showed that the subunits of SBM allergenic proteins such as glycinin and β-conglycinin were decreased by solid-state fermentation with B4 for either 24 or 48 h (Figure 4A). To verify the degradation of the allergenic protein by fermentation, western blot was performed. As shown in Figure 4B, the three allergenic proteins (75 kDa, 50 kDa, and 40 kDa) were detected in the UF-SBM samples but were either decreased or entirely eliminated in SBM after 24 h and 48 h of fermentation. Aditionally, trypsin inhibitor (17 kDa) was detected in the UF-SBM samples, while after being fermented with B4 for 24 or 48 h, the majority of the trypsin inhibitor was broken down (Figure 4B). Moreover, phytic acid analysis showthat there is a 73.3% reduction (*p* < 0.001) of phytic acid in SBM after 24 h of fermentation with B4 (Figure 4C).

### B4 exhibits undesired bacteria activity repression during SBM fermentation

**Figure 5:**
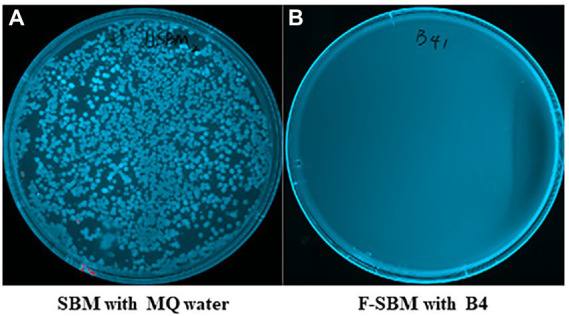
MacConkey plates assay results of gram negative bacteria. **(A)** MacConkey plate culture result of UF-SBM with sterilized MQ water at 37°C for 24 h. **(B)** MacConkey plate culture result of F-SBM with B4 at 37°C for 24 h.

To determine if B4 has antimicrobial activity that can eliminate gram-negative bacteria during fermentation, we used MacConkey plate which is selective for gram-negative bacterial growth. As shown in Figure 5, while numerous gram-negative bacteria grew on the MacConkey plates of the SBM mixed with MQ water for 24 h, there was no gram-negative bacterial growth observed in the SBM samples after 24 h of fermentation with B4.

### Changes in chemical composition between UF-SBM and F-SBM

**Figure 6:**
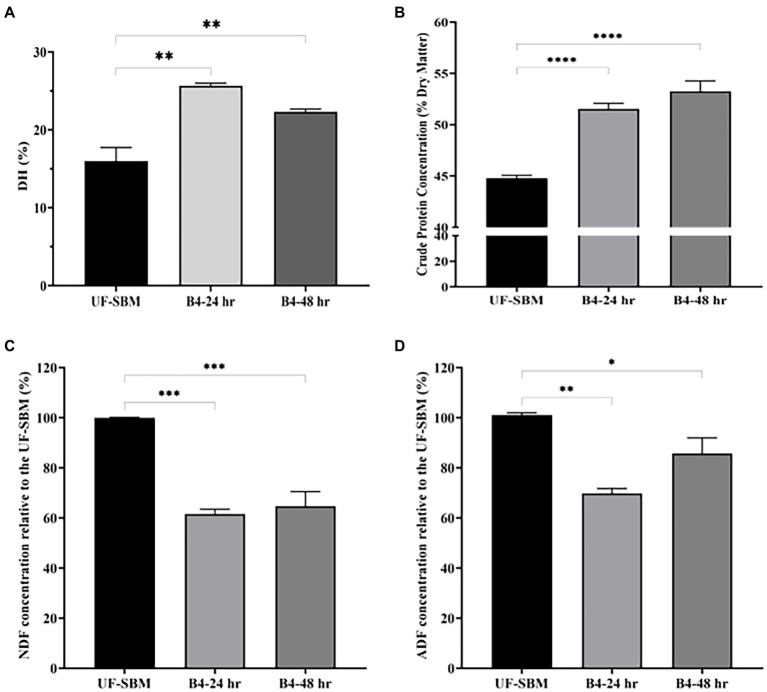
Nutritional value analysis of unfermented (UF-SBM) and fermented SBM (F-SBM). **(A)** Degree of protein hydrolysis analysis; **(B)** Crude protein concentration; **(C)** NDF concentration relative to the UF-SBM; **(D)** ADF concentration relative to the UF-SBM. UF-SBM was used as control; B4-24 h, F-SBM for 24 h; B4-48 h, F-SBM for 48 h. Data is presented as mean ±standard error of the mean (SEM). Bars with statistical significance is denoted as *p < 0.05, **p < 0.01, ***p < 0.005, ****p < 0.0001 using Ordinary one-way in ANOVA. All experiments were conducted in triplicate.

As shown in Figure 6, the results of the OPA assay displayed that the DH of SBM fermented with B4 for 24 h increases to 25.5%. No further increase was observed at 48 h of fermentation compared to the UF-SBM sample (15.9%) (Figure 6A). The crude protein concentrations of F-SBM increased significantly from 44.8% (UF-SBM) to 52.5% (*p* < 0.001, 24 h), and 54.3% (*p* < 0.001, 48 h), respectively (Figure 6B). Fermentation with B4 also significantly decreased SBM NDF and ADF (Figures 6C, D). Furthermore, fermentation with B4 significantly decreased SBM NDF and ADF values (Figures 6C, D). As showed in Figure 6C, NDF values in the F-SBM decreased by 38.4% and 35.27% after 24 h and 48 h of fermentation, respectively, (*p* < 0.001), compared to the UF-SBM. The ADF value in the F-SBM was decreased by 30.20% (*p* < 0.01) and 14.3% (*p* < 0.05) after 24 and 48 h of fermentation compared to the UF-SBM (Figure 6D). No differences were observed in the DM of F-SBM for 24 h (91.8%) and 48 h (91.4%) compared to the UF-SBM (92.4%; data not shown).

## Discussion

In this study, a potential probiotic *B. licheniformis* (B4) with phytase, protease, cellulase and xylanase activity was characterized and its fermentation potential was determined. It is found that this novel strain of *B. licheniformis* had the following unique features: firstly, B4 could tolerate low pH, and various bile salt concentration, thus ensuring that it could survive in the mammalian gastro-intestine tract; Secondly, B4 grew well at 50°C, a temperature that was not favorable to most other bacterial growth which allowed it to be the dominant bacterium when fermentation results in heat accumulation; Thirdly, B4 displays a strong ability to break down SBM anti-nutritional factors including glycinin, β-conglycinin, trypsin inhibitor and phytate when using fermentation, which in turn improved the nutritional value of SBM.

Phytase is the most commonly used exogenous enzyme additive in animal feed. It releases phosphorus from phytate to help increase the bioavailability of phosphorus and reduce the negative impacts of inorganic phosphorus output in the environment (12, 25). The fungi *Peniophora* sp. (26) and *Aspergillus* sp. (27) as well as the bacterial species *Bacillus.* spp. (9) and *Citrobacter. spp.* (28) have been widely used to produce phytasein poultry or pig feed. Additionally, *E. coli and Pichia pastoris* have also been used as hosts for the production of recombinant phytase to meet growing demands (29). In this study, a strain of phytase-producing *B. licheniformis* (B4) was selected from the screening of 37 potential probiotics isolated from camel feces. The results of phytic acid analysis show that B4 could reduce over 70% of phytic acid in the SBM after 24 h of fermentation. These findings suggest that B4 may be used to replace phytase supplementation to degrade feed phytic acid. The phytic acid degradation efficiency of B4 was higher than that of previous reported by others. In the study reported by Chen et al., a solid-state fermentation with *Aspergillus oryzae* (ATCC 9362) resulted in a 39% reduction of phytic acid (15). In another study, SBM fermentation with *Lactobacillus plantarum* decreased phytic acid by 34.13% (30). As a major anti-nutritional factor in plant sourced feed, phytic acid reduction after probiotic fermentation in many of the feed components including canola meal have been reported (23, 31, 32). Thus, B4 is an excellent candidate microorganism for feedstuff fermentation. In addition, it can reduce the cost by using B4 as a probiotic in feedstuff fermentation compared to supplement with phytase enzyme in the feed. The probiotic substances produced by B4 could also exert a synergic effect with phytase, which in turn benefits the livestock as well as develop sustainable agriculture.

Several studies have reported that bacteria including *Bacillus spp.* (3, 22), *Lactobacillus spp.* (33, 34), Fungi *Aspergillus* sp. (35, 36), and *Rhizopus sp.* (37) can be used to ferment SBM to reduce the anti-nutritional factors such as glycinin, β-conglycinin, trypsin inhibitor of SBM. Our findings suggest that B4 has robust protease, cellulase and xylanase activities, and was able to efficiently degrade glycinin, β-conglycinin, and fiber in the SBM, outlines its strong potential as a candidate microorganism for feed stuff fermentation. With these properties, B4 should be able to reduce the allergenicity and decrease anti-nutritional factors and thus improve the nutritional value of the SBM. Feedstuff processed by fermentation is rich in microorganisms that are initially used for fermentation. It would be highly desirable if these microorganisms used in fermentation can also serve as probiotics. The ability to survive in the gastrointestinal tract environment is an important requirement for the application of probiotics as additives in livestock animal feeds (38, 39). The pH range of animal stomach is around pH 2.0 to 3.0 which rarely raises above pH 3.0 even when satiated (40) and the bile salt concentration in the digestive tract is around 0.3% to 0.5% (41). In this study, B4 showed the ability to survive at pH 3.0 for 5 h, which is similar to *Bacillus licheniformis* CK1 strain isolated by Tsui Chun Hsu (42). This suggests that B4 could survive in the animal intestinal tract and has great potential as a probiotic candidate for swine and poultry production.

The temperature and undesired bacterial contamination are two important limiting factors for SBM fermentation. Previous studies have confirmed that higher or lower temperature affects not only the growth of microorganisms but also the activity of its enzymes (43). Chen et al. found that the highest phytase degradation degree was obtained at a fermentation temperature of 50°C (15). In the present study, B4 can grow rapidly over a wide range of temperatures (25 ~ 50°C) and can inhibit the growth of undesired bacteria such as gram-negative bacteria in SBM during fermentation. These properties allow more efficient, and more reproducible feedstuff fermentation.

Previous studies show that fermentation with *B. subtilis natto* isolates (44) could increase the value of the DH (15.96%) and CP (55.76%) of SBM. Similarly, CP in the SBM was increased by 8.37 and 0.34% when fermented with *B. subtilis* and *A. oryzae*, respectively, (45). Karakurt et al. (11) reported that the NDF and ADF of SBM were reduced by 43% and 40%, respectively, when fermented with *B. subtilis*. In the present study, DH contents in B4 F-SBM increased by 9.6% (15.9% to 25.5%), the crude protein was elevated from 44.8% to 54.3%, and both the NDF and the ADF were decreased significantly by 38.4% and 30.2%, respectively when compared to the unfermented SBM. This further presents B4’s potential in improving soybean meal nutrient value. Notably, the decrease of CP in F-SBM might be caused by the bacterial metabolism of amino acids produced by B4 after degradation of crude fibers (46). It could also be attributed to the large amounts of enzymes that B4 produces during fermentation, or the introduction of B4 itself which contains large amounts of bacterial cellular proteins.

In summary, we have demonstrated that the newly identified *B. licheniformis* (B4) has dual potential to be a microorganism used for both soybean meal fermentation and as a probiotic. Future animal studies using B4 fermented soybean meal are warranted to demonstrate its application potential in the swine and poultry industry and in aquaculture.

## Data availability statement

The original contributions presented in the study are included in the article/supplementary material, further inquiries can be directed to the corresponding author.

## Author contributions

JL conceived and designed the research, supervised the project, and revised the manuscript. NQ and XZ analyzed the data and wrote the manuscript. NQ, XZ, JM, MS, and K-HC performed the experiments and analyzed the data. All authors contributed to the article and approved the submitted version.

## Conflict of interest

The authors declare that the research was conducted in the absence of any commercial or financial relationships that could be construed as a potential conflict of interest.

## Publisher’s note

All claims expressed in this article are solely those of the authors and do not necessarily represent those of their affiliated organizations, or those of the publisher, the editors and the reviewers. Any product that may be evaluated in this article, or claim that may be made by its manufacturer, is not guaranteed or endorsed by the publisher.

## References

[1] Nualkul M Yuangsoi B Hongoh Y Yamada A Deevong P. Improving the nutritional value and bioactivity of soybean meal in solid-state fermentation using bacillus strains newly isolated from the gut of the termite *Termes propinquus*. FEMS Microbiol Lett. (2022) 369:fnac044. doi: 10.1093/femsle/fnac044, PMID: 35536569

[2] Zhang L Piao X. Different dietary protein sources in low-protein diets induces changes in antioxidant capacity, immunity, fecal microbiota and metabolites of weaned piglets. Anim Nutr. (2021) 8:71–81. 34977377 10.1016/j.aninu.2021.06.013 PMC8669252

[3] Akhtar N Cai HY Kiarie EG Li J. A novel *Bacillus sp.* with rapid growth property and high enzyme activity that allows efficient fermentation of soybean meal for improving digestibility in growing pigs. J Appl Microbiol. (2021) 133:3–17. 34464998 10.1111/jam.15268

[4] Beardslee TA Zeece MG Sarath G Markwell JP. Soybean glycinin G1 acidic chain shares IgE epitopes with peanut allergen Ara h 3. Int Arch Allergy Immunol. (2000) 123:299–307. doi: 10.1159/000053642, PMID: 11146387

[5] Chen YC Yu YH. Bacillus licheniformis-fermented products improve growth performance and the fecal microbiota community in broilers. Poult Sci. (2020) 99:1432–43. doi: 10.1016/j.psj.2019.10.061, PMID: 32115030 PMC7587626

[6] Dai C Hou Y Xu H Huang L Dabbour M Mintah BK. Effect of solid-state fermentation by three different bacillus species on composition and protein structure of soybean meal. J Sci Food Agric. (2022) 102:557–66. doi: 10.1002/jsfa.11384, PMID: 34145902

[7] Doyle JJ Schuler MA Godette WD Zenger V Beachy R Slightom J. The glycosylated seed storage proteins of Glycine max and *Phaseolus vulgaris*. Structural homologies of genes and proteins. J Biol Chem. (1986) 261:9228–38. doi: 10.1016/S0021-9258(18)67644-6, PMID: 3013879

[8] Zhou T Han S Li Z He P. Purification and quantification of Kunitz trypsin inhibitor in soybean using two-dimensional liquid chromatography. Food Anal Methods. (2017) 10:3350–60. doi: 10.1007/s12161-017-0902-6

[9] Zhao T Yong X Zhao Z Dolce V Li Y Curcio R. Research status of bacillus phytase. Biotech. (2021) 3:415. 10.1007/s13205-021-02964-9 PMC8377137 34485008

[10] Bayat Kohsar J Rezaii F Mahmoudnia N Ghanbari F. The effect of fermentation by *Bacillus subtilis* and *Aspergillus niger* on the nutritional value of date palm kernels. J Lives Sci Technol. (2021) 9:41–50.

[11] Karakurt Y Güvercin D Önder S Celik C Tosun R Baran B. Chemical, enzymatic, and antioxidant enrichments of full-fat soybean and sunflower meal by Bacillus subtilis (ATCC $^{®} $6633$^{TM} $) fermentation using a solid-state bioreactor. Turkish J Vet Anim Sci. (2019) 43:82–93. doi: 10.3906/vet-1803-1

[12] Velázquez-De Lucio BS Hernández-Domínguez EM Villa-García M Díaz-Godínez G Mandujano-Gonzalez V Mendoza-Mendoza B. Exogenous enzymes as Zootechnical additives in animal feed: a review. Catalysts. (2021) 11:851. doi: 10.3390/catal11070851

[13] Cheng Y-H Hsiao FS-H Wen C-M Wu C-Y Dybus A Yu Y-H. Mixed fermentation of soybean meal by protease and probiotics and its effects on the growth performance and immune response in broilers. J Appl Anim Res. (2019a) 47:339–48. doi: 10.1080/09712119.2019.1637344

[14] Yang A Zuo L Cheng Y Wu Z Li X Tong P. Degradation of major allergens and allergenicity reduction of soybean meal through solid-state fermentation with microorganisms. Food Funct. (2018) 9:1899–909. doi: 10.1039/C7FO01824J, PMID: 29536997

[15] Chen L Vadlani PV Madl RL. High-efficiency removal of phytic acid in soy meal using two-stage temperature-induced *Aspergillus oryzae* solid-state fermentation. J Sci Food Agric. (2014) 94:113–8. doi: 10.1002/jsfa.6209, PMID: 23633040

[16] Yao Y Li H Li J Zhu B Gao T. Anaerobic solid-state fermentation of soybean meal with bacillus sp. to improve nutritional quality. Front Nutr. (2021) 8:706977. doi: 10.3389/fnut.2021.706977, PMID: 34490325 PMC8418306

[17] Zhu J Wang S Gao M Zhang Y Tang Q Wang J. Preparation and in vitro evaluation of high performance fermented soybean meal using a mixture of lactobacillus plantarum, *Bacillus subtilis* and *Saccharomyces cerevisiae*. Res Square. (2019) (preprint). doi: 10.21203/rs.2.18244/v1

[18] Mun D Kyoung H Kong M Ryu S Jang KB Baek J. Effects of bacillus-based probiotics on growth performance, nutrient digestibility, and intestinal health of weaned pigs. J Anim Sci Technol. (2021) 63:1314–27. doi: 10.5187/jast.2021.e109, PMID: 34957446 PMC8672252

[19] Bernardes RD Oliveira CHD Calderano AA Ferreira RDS Dias KMM Almeida BFD. Effect of phytase and protease combination on performance, metabolizable energy, and amino acid digestibility of broilers fed nutrient-restricted diets. Rev Bras Zootec. (2022) 51:e20210211. doi: 10.37496/rbz5120210211

[20] Li Y Guo B Li C Wang W Wu Z Liu G. Isolation of a highly efficient antigenic-protein-degrading bacillus amyloliquefaciens and assessment of its safety. Animals (Basel). (2020) 10:1144. 32640687 10.3390/ani10071144 PMC7401624

[21] Sudan S Flick R Nong L Li J. Potential probiotic Bacillus subtilis isolated from a novel niche exhibits broad range antibacterial activity and causes virulence and metabolic dysregulation in Enterotoxic *E. coli*. Microorganisms. (2021):9. 34361918 10.3390/microorganisms9071483 PMC8307078

[22] Medeiros S Xie J Dyce PW Cai HY DeLange K Zhang H. Isolation of bacteria from fermented food and grass carp intestine and their efficiencies in improving nutrient value of soybean meal in solid state fermentation. J Anim Sci Biotechnol. (2018) 9:29. doi: 10.1186/s40104-018-0245-1, PMID: 29632666 PMC5885361

[23] Olukomaiya OO Fernando WC Ram Mereddy XL Sultanbawa Y. Solid-state fermentation of canola meal with *Aspergillus sojae*, *Aspergillus ficuum* and their co-cultures: effects on physicochemical, microbiological and functional properties. LWT. (2020) 127:109362. doi: 10.1016/j.lwt.2020.109362

[24] Nielsen PM Petersen D Dambmann C. Improved method for determining food protein degree of hydrolysis. J Food Sci. (2001) 66:642–6. doi: 10.1111/j.1365-2621.2001.tb04614.x

[25] Selle PH Ravindran V. Phytate-degrading enzymes in pig nutrition. Livest Sci. (2008) 113:99–122. doi: 10.1016/j.livsci.2007.05.014

[26] Pontoppidan K Pettersson D Sandberg AS. *Peniophora lycii* phytase is stabile and degrades phytate and solubilises minerals in vitro during simulation of gastrointestinal digestion in the pig. J Sci Food Agric. (2007) 87:2700–8. doi: 10.1002/jsfa.3033, PMID: 20836179

[27] Kristoffersen S Gjefsen T Svihus B Kjos NP. The effect of reduced feed pH, phytase addition and their interaction on mineral utilization in pigs. Livest Sci. (2021) 248:104498. doi: 10.1016/j.livsci.2021.104498

[28] da Silva CA Callegari MA Dias CP de Souza KL de Carvalho RH Alebrante L. Increasing doses of bacterial Phytase (Citrobacter braakii) improves performance and carcass characteristics of pigs in growing and finishing phases. Animals (Basel). (2022) 12:2552. 36230293 10.3390/ani12192552 PMC9558933

[29] Liu L Yang H Shin H-D Chen RR Li J Du G. How to achieve high-level expression of microbial enzymes: strategies and perspectives. Bioengineered. (2013) 4:212–23. doi: 10.4161/bioe.24761, PMID: 23686280 PMC3728192

[30] Istiqomah L Damayanti E Suryani A Susilawati I. Bio-modified soybean meal as a new protein source for food In: IOP conference series: Materials science and engineering. Philadelphia, PA: IOP Publishing (2021). 012010.

[31] Al-Asheh S Duvnjak Z. Phytase production and decrease of phytic acid content in canola meal by *Aspergillus carbonarius* in solid-state fermentation. World J Microbiol Biotechnol. (1995) 11:228–31. doi: 10.1007/BF00704655, PMID: 24414509

[32] Brinch-Pedersen H Hatzack F Sorensen LD Holm PB. Concerted action of endogenous and heterologous phytase on phytic acid degradation in seed of transgenic wheat (*Triticum aestivum* L.). Transgenic Res. (2003) 12:649–59. doi: 10.1023/B:TRAG.0000005113.38002.e1, PMID: 14713194

[33] Cheng Y-H Su L-W Horng Y-B Yu Y-H. Effects of soybean meal fermented by lactobacillus species and *Clostridium butyricum* on growth performance, diarrhea incidence, and fecal bacteria in weaning piglets. Ann Anim Sci. (2019b) 19:1051–62. doi: 10.2478/aoas-2019-0042

[34] de Oliveira NS Ha N da Cunha L Cipriani LA Neto AT Skoronski E. Fermentation of soybean meal with lactobacillus acidophilus allows greater inclusion of vegetable protein in the diet and can reduce Vibrionacea in the intestine of the south American catfish (*Rhamdia quelen*). Animals (Basel), (2022). 12:690. doi: 10.3390/ani12060690, PMID: 35327087 PMC8944494

[35] Naing HTH Swe KH Muu KS. Solid state fermentation of soybean meal with *Aspergillus niger* for upgrading nutritive values. Int J Innov Educ Res. (2019) 7:374–81.

[36] Kohsar JB Rezaii F Mahmoudnia N Ghanbari F. The effect of fermentation by *Bacillus subtilis* and *Aspergillus niger* on the nutritional value of date palm kernels. J Lives Sci Technol. (2021) 9:41–50.

[37] Mukherjee R Chakraborty R Dutta A. Role of fermentation in improving nutritional quality of soybean meal - a review. Asian-Australas J Anim Sci. (2016) 29:1523–9. 26954129 10.5713/ajas.15.0627 PMC5088370

[38] Doeschate KIT Coyne VE. Improved growth rate in farmed *Haliotis midae* through probiotic treatment. Aquaculture. (2008) 284:174–9. doi: 10.1016/j.aquaculture.2008.07.018

[39] Widanarni Widagdo P Wahjuningrum D. Oral application of probiotic, prebiotic, and synbiotic in Pacific white shrimp (*Litopenaeus vannamei*) challenged with *Vibrio harveyi*. Jurnal Akuakultur Indonesia. (2012) 11:54–63.

[40] Beasley DE Koltz AM Lambert JE Fierer N Dunn RR. The evolution of stomach acidity and its relevance to the human microbiome. PLoS One. (2015) 10:e0134116. doi: 10.1371/journal.pone.0134116, PMID: 26222383 PMC4519257

[41] Andriani Y Safitri R Rochima E Fakhrudin SD. Characterization of Bacillus subtilis and B. licheniformis potentials as probiotic bacteria in Vanamei shrimp feed (*Litopenaeus vannamei* Boone, 1931). Nusantara Biosci. (2017) 9:188–93. doi: 10.13057/nusbiosci/n090214

[42] Hsu TC Yi PJ Lee TY Liu JR. Probiotic characteristics and zearalenone-removal ability of a bacillus licheniformis strain. PLoS One. (2018) 13:e0194866. doi: 10.1371/journal.pone.0194866, PMID: 29641608 PMC5895015

[43] Wang YH Feng JT Zhang Q Zhang X. Optimization of fermentation condition for antibiotic production by *Xenorhabdus nematophila* with response surface methodology. J Appl Microbiol. (2008) 104:735–44. doi: 10.1111/j.1365-2672.2007.03599.x, PMID: 17953686

[44] Zhang Y Ishikawa M Koshio S Yokoyama S Dossou S Wang W. Optimization of soybean meal fermentation for aqua-feed with Bacillus subtilis natto using the response surface methodology. Fermentation. (2021) 7:306. doi: 10.3390/fermentation7040306

[45] Teng D Gao M Yang Y Liu B Tian Z Wang J. Bio-modification of soybean meal with Bacillus subtilis or *Aspergillus oryzae*. Biocatal Agric Biotechnol. (2012) 1:32–8. doi: 10.1016/j.bcab.2011.08.005

[46] Ali N Muktiani A Pangestu E. Quality of crude protein and crude fibre wafer complete feed based on rice straw fermented with effective microorganism 4 (EM-4) In: IOP conference series: Earth and environmental science. Philadelphia, PA: IOP Publishing (2020). 012028.

